# Cortical and Subcortical Changes in MEG Activity Reflect Parkinson’s Progression over a Period of 7 Years

**DOI:** 10.1007/s10548-023-00965-w

**Published:** 2023-05-08

**Authors:** Lennard I. Boon, Arjan Hillebrand, Menno M. Schoonheim, Jos W. Twisk, Cornelis J. Stam, Henk W. Berendse

**Affiliations:** 1 Department of Neurology, Amsterdam UMC, Vrije Universiteit, Amsterdam, The Netherlands; 2 Department of Clinical Neurophysiology and Magnetoencephalography Center, Amsterdam UMC, Vrije Universiteit, Amsterdam, The Netherlands; 3 Department of Anatomy and Neurosciences, Amsterdam UMC, Vrije Universiteit, Amsterdam, The Netherlands; 4 Department of Epidemiology and Biostatistics, Amsterdam UMC, Vrije Universiteit, Amsterdam, The Netherlands

**Keywords:** Magnetoencephalography, Resting-state, Biomarker, Longitudinal, Subcortical brain regions, Spectral power

## Abstract

In this study of early functional changes in Parkinson’s disease (PD), we aimed to provide a comprehensive assessment of the development of changes in both cortical and subcortical neurophysiological brain activity, including their association with clinical measures of disease severity. Repeated resting-state MEG recordings and clinical assessments were obtained in the context of a unique longitudinal cohort study over a seven-year period using a multiple longitudinal design. We used linear mixed-models to analyze the relationship between neurophysiological (spectral power and functional connectivity) and clinical data. At baseline, early-stage (drug-naïve) PD patients demonstrated spectral slowing compared to healthy controls in both subcortical and cortical brain regions, most outspoken in the latter. Over time, spectral slowing progressed in strong association with clinical measures of disease progression (cognitive and motor). Global functional connectivity was not different between groups at baseline and hardly changed over time. Therefore, investigation of associations with clinical measures of disease progression were not deemed useful. An analysis of individual connections demonstrated differences between groups at baseline (higher frontal theta, lower parieto-occipital alpha2 band functional connectivity) and over time in PD patients (increase in frontal delta and theta band functional connectivity). Our results suggest that spectral measures are promising candidates in the search for non-invasive markers of both early-stage PD and of the ongoing disease process.

**Supplementary Information:**

The online version contains supplementary material available at 10.1007/s10548-023-00965-w.

## Introduction

Parkinson’s disease is a progressive neurodegenerative disease characterized by classical motor symptoms as well as a wide range of non-motor symptoms, among which cognitive decline (Chaudhuri et al. 2006). The pathological hallmark of Parkinson’s disease is the deposition of alpha synuclein in the brain, which initially mainly affects the brainstem, including the neurons of the nigrostriatal dopamine system, and extends to widespread cortical brain regions in more advanced disease stages (Braak et al. 2003). This pathology affects the brain function, although its mechanism is as yet unknown.

Magnetoencephalography (MEG) can be used to measure brain activity with high temporal and good spatial resolution (Baillet 2017; Litvak et al. 2021). Changes in neurophysiological measures of cortical brain activity such as spectral slowing and loss of interactions between brain regions, i.e. functional connectivity, are well-established phenomena in Parkinson’s disease (Bosboom et al. 2006, 2009b; Caviness et al. 2007, 2016; Hassan et al. 2017; Stoffers et al. 2007). These changes correlate with clinical measures of disease progression (Geraedts et al. 2018; Olde Dubbelink et al. 2013a, 2013b; Yassine et al. 2022) and can have predictive value for cognitive deterioration, such as the conversion to Parkinson’s disease dementia (Arnaldi et al. 2017; Klassen et al. 2011; Olde Dubbelink et al. 2014b). Hence, neurophysiological patterns hold promise as biomarkers of the degenerative process in Parkinson’s disease, for instance for prognosis or the assessment of treatment effects of future disease-modifying therapies.

Already at the earliest Parkinson’s disease stages, slowing of oscillatory brain activity and functional connectome changes have been demonstrated (De Micco et al. 2021; Stoffers et al. 2007). As nigrostriatal changes lie at the heart of early-stage Parkinson’s disease (i.e. motor) symptoms, one would expect functional changes in subcortical brain regions as well. Previous neurophysiological studies have focused on cortical brain regions, with the exception of local field potentials recorded from the subthalamic nucleus in deep brain stimulation-treated patients. In recent years, increasing evidence suggests the feasibility to project MEG-signals onto other subcortical brain regions (Boon et al. 2017; Hillebrand and Barnes 2005; Hillebrand et al. 2012, 2016a, 2005; Pizzo et al. 2019), which allows for a reliable measurement of functional properties of these subcortical brain areas.

Previous longitudinal studies in Parkinson’s disease using EEG or MEG had a follow-up duration of approximately four years (Caviness et al. 2015; Olde Dubbelink et al. 2013a, 2013b; Yassine et al. 2022) or were performed in moderately advanced Parkinson’s disease patients (average disease duration of 8.5 years at baseline in that study) (Caviness et al. 2015). Hence, the full picture of the neurophysiological changes that occur throughout the course of Parkinson’s disease is currently lacking. Ideally, one would follow a cohort of Parkinson’s disease patients from disease onset up to a disease duration of 15–20 years. In our study we have met this aim by using a so-called ‘multiple longitudinal design’ in which we included patients with different baseline disease durations (ranging from early-stage, drug-naive patients to patients with a disease duration of 13 years) in combination with a seven year follow-up duration. We recently demonstrated the feasibility of combining data recorded longitudinally on two different MEG systems in healthy controls, by analyzing the MEG data in source-space and excluding the gamma band (Boon et al. 2021). In this study, the spectral power and functional connectivity results remained stable over time. This allowed us to combine the MEG data recorded in a Parkinson’s disease cohort over a period of seven years using two different recording systems (CTF at baseline and follow-up 1; Elekta Vectorview at follow-up 2).

In the present study, we assessed MEG-based measures of spectral power and functional connectivity (the corrected amplitude envelope correlation (AEC-c)) at three time points in a cohort that initially included 61 Parkinson’s disease patients and 16 healthy controls. We hypothesized to find neurophysiological changes in subcortical brain regions in de novo Parkinson’s disease, and expected these to be more prominent than the changes in cortical brain regions. In addition, we expected longitudinal neurophysiological changes in subcortical brain regions to be associated most strongly with Parkinson’s disease-related motor impairments and neurophysiological changes in (mainly posterior) cortical brain regions to be associated most strongly with cognitive decline (Boon et al. 2017; Olde Dubbelink et al. 2014b; Scheijbeler et al. 2022).

## Materials and Methods

### Participants

**Fig. 1:**
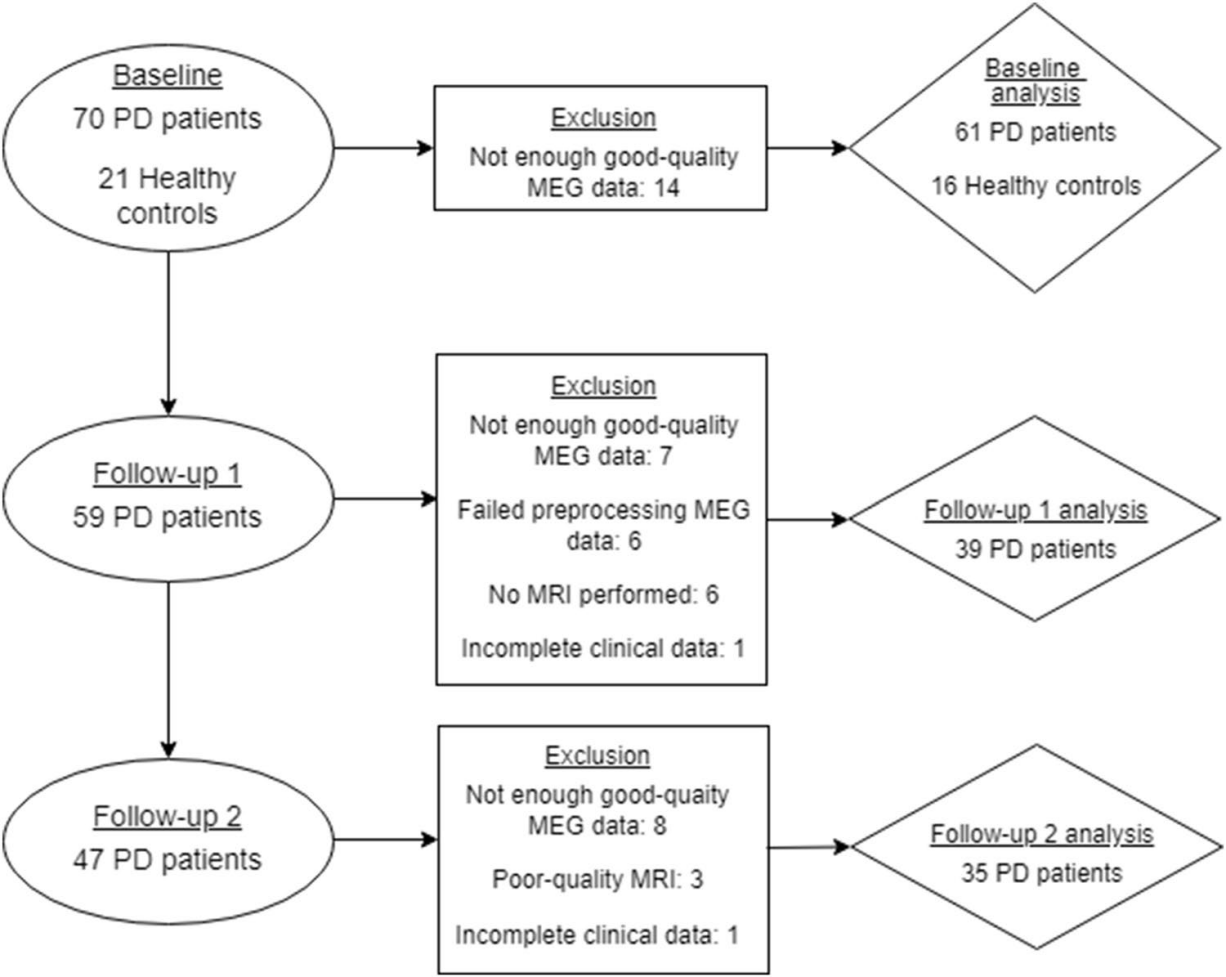
Flowchart of patient inclusion. *PD* Parkinson’s disease, *MEG* magnetoencephalography

At baseline, 70 non-demented patients with idiopathic Parkinson’s disease (disease duration 0–13 years, including 18 early-stage drug-naïve (de novo) patients) and 21 healthy controls (age-matched to the de novo patients) were consecutively approached and included in this multiple longitudinal study at Amsterdam UMC from April 2003 to March 2006 (Boon et al. 2017; Olde Dubbelink et al. 2014a, 2014b, 2013a, 2013b; Stoffers et al. 2007). The inclusion and exclusion criteria have previously been described in (Stoffers et al. 2007). Patients underwent motor and cognitive assessments, as well as MEG and MRI recordings at three time points (BL and two follow-up visits scheduled approximately 4 and 7 years later). Only data of the BL study visit were used in case of the healthy controls (HCs), part of the follow-up data has previously been used (Boon et al. 2021). Figure 1 shows the number of participants at each time point, as well as the number of participants included in the final analysis (and the reason for exclusion). 61 patients (including 17 de novo patients) and 16 HCs were analyzed at baseline (BL), 39 patients at follow-up 1 (FU1), and 35 patients at follow-up 2 (FU2).

All participants gave written informed consent to the research protocol, which was approved by the medical ethical committee of Amsterdam UMC, location VU University Medical Center (Amsterdam, The Netherlands). Ethics review conformed to the Helsinki declaration. All recordings/assessments were carried out in accordance with relevant guidelines and regulations. Reporting of this study meets the STROBE guidelines (Vandenbroucke et al. 2007).

### Participants Characteristics

Disease duration was calculated on the basis of the patients’ estimation of the onset of the classical Parkinson’s disease motor symptoms. Educational level was determined on the basis of the International Standard Classification of Education (ISCED).(UNESCO 1997) Unified Parkinson’s Disease Rating Scale motor ratings (UPDRS-III) (Fahn S 1987) were obtained in the ‘ON’ medication state by a trained physician (with the exception of 17 de novo Parkinson’s disease patients at baseline who were not on dopaminergic medication yet). For each patient, the Hoehn and Yahr stage was determined (Hoehn and Yahr 1967). The total dose of dopamine replacement therapy was converted to a so-called levodopa equivalent daily dose (LEDD) as described previously (Olde Dubbelink et al. 2013a). Levodopa was always used in combination with a peripheral decarboxylase inhibitor. Two patients were using rivastigmine at the time of FU1 and one patient at the time of FU2. Global cognitive function was assessed using the Cambridge Cognitive Examination (CAMCOG) scale (Roth et al. 1986). Conversion to PD dementia was assessed using the clinical criteria recommended by the Movement Disorders Society Task Force (Dubois et al. 2007).

### Data Acquisition

During all recordings, patients were on their usual dose of dopaminergic medication (note that the 17 de novo cases did not use dopaminergic medication at BL). BL and FU1 MEG data were acquired using a 151-channel whole-head MEG system (CTF systems Inc., Vancouver, Canada) in an eyes-closed resting-state condition for five minutes while participants were seated inside a magnetically shielded room. Sample rates of 312.5 (BL) and 625 Hz (FU1) were used during recordings. At the beginning and end of each recording the head position relative to the coordinate system of the helmet was assessed by leading small currents through three head position indicator (HPI) coils attached to the left and right pre-auricular points and the nasion. For more details on the recordings, see previous publications from our group (Boon et al. 2021; Olde Dubbelink et al. 2013a).

FU2 MEG data were recorded using a 306-channel Vectorview system (Elekta Neuromag, Oy, Helsinki, Finland) in an eyes-closed resting-state condition for five minutes in a supine position, with a sample rate of 1250 Hz. The head position relative to the MEG sensors was recorded continuously using the signals of four HPI coils. The coil positions were digitized before each recording, as well as the outline of the patient’s scalp (~ 500 points), using a 3D digitizer (Fastrak, Polhemus, Colchester, VT, USA).

Structural T1-weighted MR imaging was performed at all three time points (BL; 1.0 T, Impact, Siemens, Erlangen, Germany; FU1 and FU2; 3.0 T, GE Signa HDxt, Milwaukee, WI, USA). In preparation for the MR imaging at BL and FU1, vitamin E capsules were placed at the same anatomical landmarks where the three HPI coils had been placed during MEG-registration.

### Data Preprocessing

Standard implementations of the analysis pipeline were used for both MEG systems as described below. FU1 MEG data were downsampled to 312.5 Hz to obtain the same sample rate as at BL. MEG channels that were malfunctioning, for example due to excessive noise, were identified based on visual inspection and not included in the further analysis (all by the same observer KTEOD; mean number of excluded channels: BL/FU1 2.4, range 2–7; FU2 6.7, range 2–11). BL and FU1 data were split into epochs (13.11 s, 4096 samples) and epochs containing artefacts were discarded (BL mean 2.5, range 0–11; FU1 mean 2.8, range 0–12). In addition, the temporal extension of Signal Space Separation (tSSS) in MaxFilter software (Elekta Neuromag Oy, version 2.2.15) was applied to FU2 data to remove artefacts (Taulu and Hari 2009; Taulu and Simola 2006), using a sliding window of 10 s and a subspace correlation-limit of 0.9.

Participants’ MEG data were co-registered to their structural MRIs using in-house software, in case of BL/FU1 through identification of the same anatomical landmarks (left and right pre-auricular points and nasion; estimated co-registration error < 6 mm) in both modalities, and in case of FU2 through a surface-matching procedure, with an estimated resulting accuracy of 4 mm (Whalen et al. 2008). For all three time points, the automated anatomical labelling (AAL) atlas was used to label the voxels in 78 cortical and 12 subcortical regions of interest (ROIs) in the subjects’ co-registered MRI using SPM (Gong et al. 2009; Tzourio-Mazoyer et al. 2002). In order to reconstruct a single time-series of neuronal activity for a ROI (Hillebrand et al. 2012) we used each ROI’s centroid as representative for that ROI (Hillebrand et al. 2016b). A scalar beamformer was used to reconstruct beamformer weights for each centroid using broadband data (0.5–48 Hz) (Hillebrand and Barnes 2005; Hillebrand et al. 2005) (Synthetic Aperture Magnetometry (SAM)) (Vrba and Robinson 2002) for BL and FU1, and Elekta’s beamformer implementation, a scalar beamformer comparable to SAM (version 2.2.15; Elekta Neuromag Oy) for FU2). Mean length of broadband data was 231 s (range 157 – 432 s) for BL, 252 s (range 130 – 328 s) for FU1, and 313 s (range 296 – 495 s) for FU2. Broadband data were projected through the normalized (Cheyne et al. 2007) beamformer weights for each centroid of the 90 ROIs. For further details on the projection of MEG data to source-space, see our previous publication on the healthy controls of this cohort (Boon et al. 2021). Subsequently, FU2 time-series were downsampled (4x, in order to obtain the same sample rate as at BL) and split into epochs of 4096 samples (13.11 s) as well.

Ten artefact and drowsiness-free epochs were selected per subject at each time point by visual analysis (KTEOD/LIB). For frequency band specific analyses, epochs were filtered in five canonical frequency bands (delta (0.5–4 Hz), theta (4–8 Hz), alpha1 (8–10 Hz), alpha2 (10–13 Hz, as the distinction between alpha1 and alpha2 oscillations may have functional significance (Klimesch et al. 2005)), and beta (13–30 Hz). We excluded the gamma band, as we previously demonstrated in healthy subjects that gamma power values were not stable between the two different MEG systems, possibly due to a difference in the handling of artefacts (Boon et al. 2021).

### Data Analysis

**Fig. 2:**
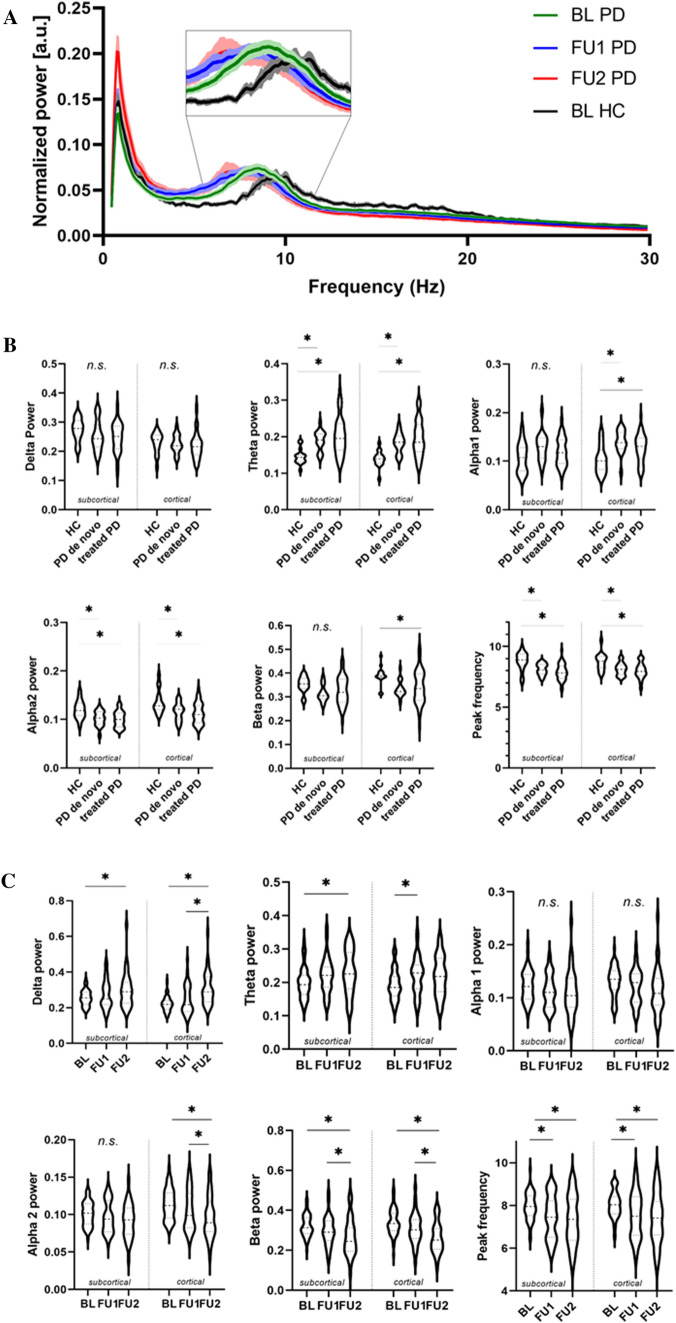
Power spectral analysis. **A** Whole-group normalized power spectra at three time points, including the healthy controls (HC) at baseline (BL). Spectral power between 0.5 and 30 Hz was averaged over all brain regions. **B** Violin plots summarizing the baseline comparison between three groups; de novo PD patients, more advanced treated PD patients and HCs. A significance level of 0.05/6 (Bonferroni correction) was applied. **C** Violin plots summarizing the longitudinal analysis. Statistical testing was performed using linear mixed-models. A significance level of 0.05/6 (Bonferroni correction) was applied. *n.s.* non-significant, *HC* healthy control, *FU1* follow-up 1, *FU2* follow-up 2

Spectral and functional connectivity analyses were performed using in-house software (BrainWave, version 0.9.152.12.26; CJS, available from http://home.kpn.nl/stam7883/brainwave.html). We created group-averaged power spectra (0.5–30 Hz) for each time point separately, normalized the spectra using the area under the curve (Fig. 2A), and determined the peak frequency within the 4–13 Hz frequency range (spectral resolution of 0.076 Hz). Furthermore, for each ROI, we estimated frequency band specific relative power (relative to the total spectral power between 0.5 and 30 Hz) and functional connectivity with the rest of the brain.

We estimated functional connectivity using the AEC-c, an implementation of the AEC (Brookes et al. 2012; Bruns et al. 2000) corrected for volume conduction/field spread, using a symmetric orthogonalisation procedure (Brookes et al. 2012; Hipp et al. 2012) applied to each epoch: To adjust for any negative correlations, 1 was added to the raw AEC values and subsequently divided by 2, leading to values between 0 and 1, and with 0.5 indicating absence of functional connectivity. The AEC-c was calculated for all possible pairs of ROIs, leading to a 90*90 adjacency matrix. AEC-c values were subsequently averaged per ROI. This resulted in a single AEC-c value per ROI (per frequency band), reflecting the mean functional connectivity of that region with the rest of the brain.

Next, both relative power values and functional connectivity values were averaged for i) all cortical brain regions (regions 1–78; ii) all subcortical brain regions (regions 79–90, see Supplementary Table 1 for definitions and order of the regions). Results of 10 epochs were averaged per patient.

### Statistical Analysis

#### Patient Characteristics

The baseline composition of the study cohort was statistically tested as follows: Age, disease duration, UPDRS-III score, LEDD total dose, and CAMCOG were tested using independent sample *t-*tests. Sex and HY score were tested using Chi-square tests and ISCED using Fisher’s exact test. The cohorts studied at BL, FU1 and FU2 showed differences due to drop-out of patients. Hence, our analysis can be considered as a missing data analysis. The group composition over time was therefore not statistically tested.

#### Spectral Power and Functional Connectivity: Baseline Comparison

First, we analyzed spectral power and functional connectivity averaged over (i) all cortical brain regions and (ii) all subcortical brain regions, separately for HCs, de novo patients and treated (more advanced) patients. Next, we performed linear mixed-models separately for the cortical and subcortical brain regions, with a grouping variable (HCs, de novo and more advanced treated PD patients; represented by dummy variables) and the covariates age, gender and ISCED (dichotomized at 3).

In order to explore whether neurophysiological characteristics of subcortical brain regions differed more from HCs than those of cortical brain regions, we created linear mixed-models with the neurophysiological measure as dependent variable, the grouping variable (control or de novo PD patient), location variable (cortical or subcortical), the interaction between the group and location variables, as well as the covariates age, gender and ISCED (dichotomized).

As an additional analysis, we explored whether functional connectivity between individual brain regions (connections) was significantly different between healthy controls and the two PD groups. Therefore, we applied permutation tests (N = 50,000; p < 0.05) on all 90*90 individual, with an FDR correction for the number of connections.

#### Spectral Power and Functional Connectivity: Longitudinal Changes

We used linear mixed-models to evaluate the longitudinal changes in spectral power and functional connectivity in Parkinson’s disease patients. Linear mixed-models can account for the dependency of the observations within the patient and the fact that not all patients had undergone a (complete) study visit at all three time points. In the linear mixed-models the time variable (BL, FU1, FU2) was treated as a categorical variable and represented by dummy variables. Age at baseline, disease duration at baseline, gender, ISCED (dichotomized), and LEDD were included in the model as covariates. MEG-machine was not included as covariate here, as it was redundant because of the inclusion of the time variable. Relative spectral power (per frequency band; delta to beta band), peak frequency, and functional connectivity (per frequency band; functional connectivity of one brain region with the rest of the brain) were each averaged over cortical and subcortical brain regions separately and were used as dependent variables.

Next, we explored whether the strength of the functional connections significantly changed over time (BL to FU1) for the PD group. Again, we applied permutation tests (N = 50,000; p < 0.05) on all individual connections, with an FDR correction for the number of connections.

#### Relationship Between Spectral Power Measures and Clinical Measures of Disease Progression

We analyzed the relationship between the longitudinal courses of the spectral measures and global measures of cognitive (CAMCOG) and motor (UPDRS-III) function using linear mixed-models. CAMCOG and UPDRS-III were treated as dependent variables, spectral measures (delta to beta relative power and peak frequency) as independent variables. Age at baseline, disease duration at baseline, gender, ISCED (dichotomized; only included in case of analyses involving CAMCOG), recording system (CTF versus Elekta), and LEDD were included in the model as covariates. Neurophysiological measures of i) cortical brain regions and ii) subcortical brain regions were used as independent variables in separate analyses. Due to collinearity of the neurophysiological measures, it was not possible to combine them in a single linear mixed-model.

Statistical analyses were performed using the IBM SPSS Statistics 26 software package (IBM Corporation, New York, USA). A significance level of 0.05/6 was applied in the baseline comparisons and the analyses on longitudinal changes, as we applied Bonferroni correction for the number of comparisons (comparisons between three groups (HC’s, early and more advanced PD patients) or three time points and two groups of brain regions (subcortical and cortical)), separately for each frequency band). In the other analyses, a significance level of 0.05 was applied. In the linear mixed-models, a random intercept was used. Supplementary Fig. 1 gives an overview of the statistical tests performed in this study.

## Results

### Patient Characteristics

Participant characteristics and details on statistics are summarized in Table 1. The HCs were age-matched to the de novo PD patients, the more advanced (treated) patients were significantly older. All 17 de novo patients were included in the longitudinal analysis, of which 9 completed all three study visits. Twenty-two patients had a ‘complete’ dataset and were analyzed at three time points. HCs had significantly better CAMCOG scores than both Parkinson’s disease groups (Table 1A). At FU1, three patients fulfilled the criteria for Parkinson’s disease dementia and at FU2 six patients fulfilled these criteria. The composition of the cohorts studied at BL, FU1 and FU2 are demonstrated in Table 1B.

**Table 1:** Participant characteristics

(A)	HC (n = 16)	De novo PD (n = 17)	Treated PD patients (n = 44)	Statistics HC- De novo PD	Statistics HC- Treated PD
Sex (M/F)	10/6	12/5	21/23	X^2^(31) = 0.243; *p* = .622	X^2^(31) = 1.02; *p* = .387
Age baseline (years)	58.9 (6.6)	60.6 (7.9)	64.1 (0.9)	t(31) = 0.670; *p* = .510	
	**t(58) = 2.80; *p* = .006**				
Disease duration baseline (years)	n/a	0.94 (0.42)	7.1 (3.0)	n/a	n/a
ISCED (1/2/3/4/5/6)	0/5/3/0/8/0	0/5/4/0/8/0	1/20/10/4/9/0	Fisher’s exact test: *p* = 1.00	
	Fisher’s exact test: *p* = 0.226				
UPDRS-III	n/a	13.2 (6.3)	13.4 (5.2)	n/a	n/a
LEDD total dose	n/a	n/a	524 (394)	n/a	n/a
HY stage (0/1/1.5/2.0/2.5/3/4/5)	n/a	0/8/1/7/1/0/0/0	0/6/1/25/12/0/0/0	n/a	n/a
CAMCOG	99.1 (3.6)	95.8 (5.1)	94.6 (5.0)	**t(31) = 2.09; *p* = .045**	**t(58) = 3.30; *p* = .002**

(B)	PD BL (n = 61)	FU1 (n = 39)	FU2 (n = 35)
Sex (M/F)	33/28	25/14	19/16
Age baseline (years)	63.2 (6.8)	61.6 (6.6)	62.1 (6.0)
Disease duration baseline (years)	5.35 (3.8)	4.66 (3.7)	4.92 (3.7)
ISCED (1/2/3/4/5/6)	1/25/14/4/17/0	1/12/12/1/12/1	1/12/10/2/10/0
UPDRS-III	13.4 (5.5)	27.3 (8.7)	32.9 (9.7)
LEDD total dose	382 (409)	746 (435)	1128 (520)
HY stage (0/1/1.5/2.0/2.5/3/4/5)	0/14/2/32/13/0/0/0	1/0/0/12/20/6/0/0	0/0/0/13/10/10/1/1
CAMCOG	94.9 (5.0)	92.8 (8.6)	89.9 (14.0)
Parkinson’s disease dementia	0	3	6

(A) Cross-sectional analysis. (B) Longitudinal analysis in PD patients. Numbers are expressed as mean (standard deviation)

*PD* Parkinson’s disease, *M* male, *F* female, *ISCED* International Standard Classification of Education, *n/a* not applicable, *UPDRS-III* motor part of Unified Parkinson’s Disease Rating Scale, *LEDD* levodopa equivalent daily dose, HY stage (Hoehn and Yahr stage), *CAMCOG* Cambridge Cognitive examination, *n/a* not applicable, *BL* baseline, *FU1* follow-up 1, *FU2* follow-up 2

### Spectral Power

Figure 2A shows a global normalized power spectrum, group-averaged per time point, in which de novo and treated Parkinson’s disease patients were combined. At BL, the peak frequency was significantly lower in the Parkinson’s disease group than in the HC group (Fig. 2B). The peak frequency also significantly decreased further over time in the Parkinson’s disease group (Fig. 2C).

Looking at spectral power, the baseline analysis (Fig. 2B) demonstrates significantly higher relative theta band power in both Parkinson’s disease groups compared with healthy controls, both for cortical and subcortical brain regions. Relative alpha2 power and peak frequency were significantly lower in the Parkinson’s disease groups, both for cortical and subcortical brain regions. In addition, only for the cortical brain regions, alpha1 band power was significantly higher (all Parkinson’s disease patients) and beta band power significantly lower (treated patients). There were no significant differences between the de novo patients and the treated patients. In addition, using linear mixed-models, we found that de novo PD patients deviated from controls stronger for cortical than for subcortical brain regions for alpha1 and beta band power (*p* = 0.028 and 0.008, respectively).

Over time, the pattern of slowing further developed as we found increases in relative delta and theta power, and decreases in relative alpha2 and beta power. The magnitude of these changes was comparable between cortical and subcortical brain regions (Fig. 2C; see Supplementary Table 2 for statistics).

### Functional Connectivity

There were no significant baseline group differences in the whole-brain functional connectivity analysis (Supplementary Fig. 2A). We also observed no significant longitudinal changes in whole-brain functional connectivity, except for a significant increase in subcortical beta band functional connectivity between FU1 and FU2 (Supplementary Fig. 2B). Because of the scarcity of results in the whole-brain analyses, functional connectivity was not used as input for the associations with clinical measures of disease severity.

**Fig. 3:**
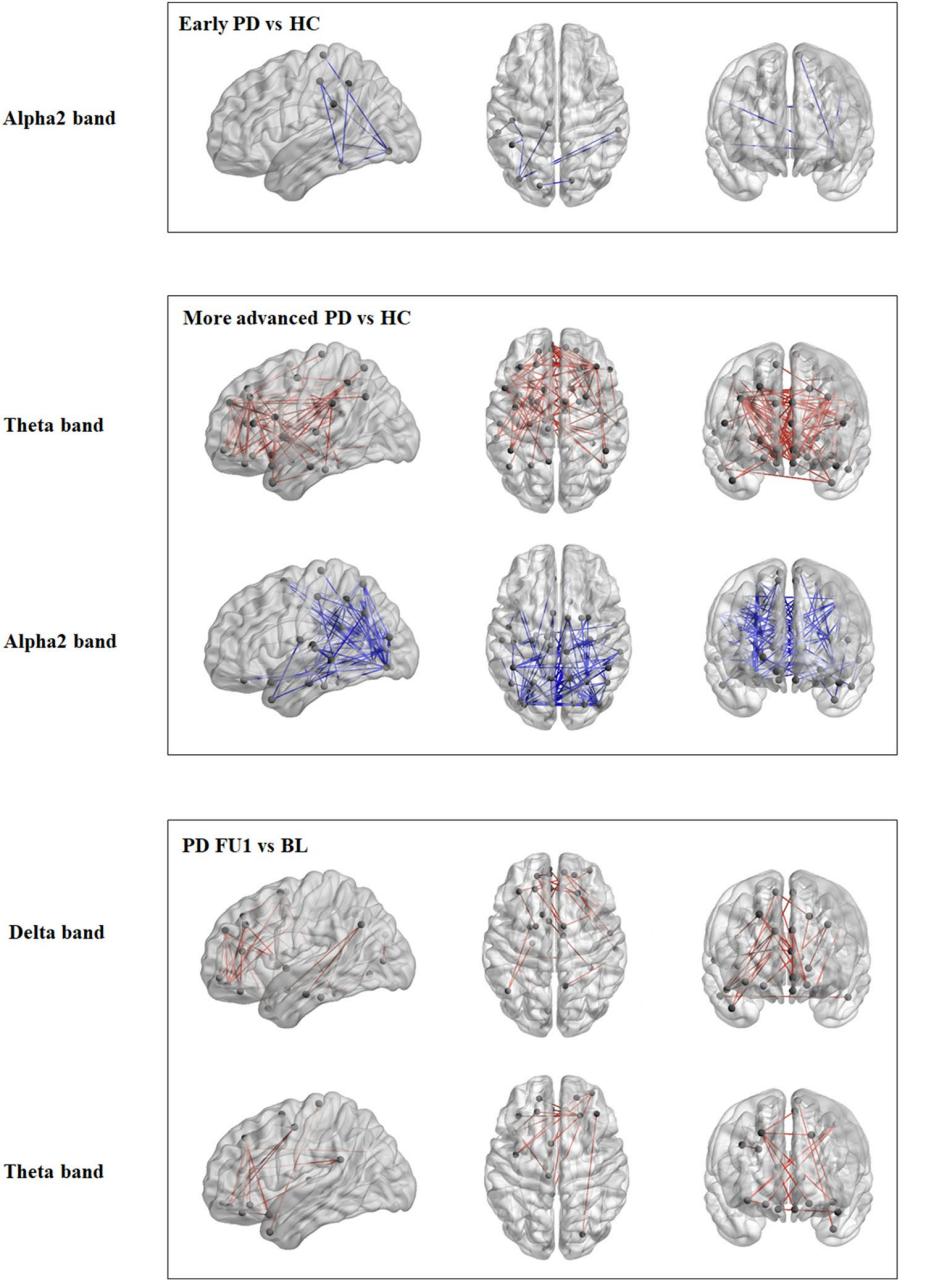
Functional connectivity: Connection-specific analysis. Distribution of significant differences in functional connectivity between two individual brain regions. Differences are displayed on a standard brain viewed from (left to right) left lateral, top, and frontal (Xia et al. 2013). Red represents a significant increase in functional connectivity, blue a decrease. In the top two panels we compared two groups at baseline, in the lowest panel we compared two time points in the PD group. PD, Parkinson’s disease; HC, healthy control; BL, baseline; FU1, follow-up 1

In the connection-specific analysis (Fig. 3) we found significantly higher theta band functional connectivity for the more advanced PD patients compared with HCs, mainly for the frontal connections. In addition, there was significantly lower alpha2 band functional connectivity of parieto-occipital connections for the PD group (both early and more advanced patients) compared with HCs.

In the longitudinal analysis of the PD group, between BL and FU1, we found significant increases in delta and theta band functional connectivity for frontal connections.

### Relationship Between Neurophysiological Parameters and Clinical Measures of Disease Progression

We analyzed the longitudinal relationship between the spectral measures (delta to beta band relative power, peak frequency) and scores on the CAMCOG and UPDRS-III, separately for cortical and subcortical brain regions.

Both for cortical and subcortical brain regions, higher relative delta power was significantly associated with worse performance on CAMCOG. In addition, lower relative alpha1, alpha2, and beta power, as well as lower peak frequency, all in cortical and subcortical areas, were associated with worse performance on CAMCOG (Table 2). Conversely, higher (cortical) relative delta power and (cortical and subcortical) theta power were associated with higher scores on the UPDRS-III. In addition, lower relative (cortical) alpha2, lower relative (cortical and subcortical) beta power, and lower peak frequency (cortical and subcortical) were associated with worse motor performance (i.e. higher scores on UPDRS-III).

**Table 2:** Longitudinal associations between spectral measures and clinical measures of disease severity

CAMCOG	Subcortical brain regions			Cortical brain regions		
	Estimated regression coefficient	95% CI	*p* value	Estimated regression coefficient	95% CI	*p* value
*Relative power*						
Delta	**-75.1**	**-92.1 to -58.1**	** < .001**	**-79.0**	**-96.9 to -61.0**	** < .001**
Theta	-11.7	-43.3 to 19.8	.463	-21.2	-54.0 to 11.5	.201
Alpha1	**115.3**	**71.9 to 158.8**	** < .001**	**112.5**	**68.9 to 156.1**	** < .001**
Alpha2	**201.4**	**123.1 to 279.7**	** < .001**	**166.0**	**102.6 to 299.5**	** < .001**
Beta	**52.4**	**30.9 to 74.0**	** < .001**	**51.2**	**28.9 to 73.5**	** < .001**
Peak frequency	**4.2**	**2.6 to 5.8**	** < .001**	**4.5**	**2.9 to 6.1**	** < .001**

UPDRS-III	Subcortical brain regions Estimated regression coefficient 95% CI			Cortical brain regions		
			*p* value	Estimated regression coefficient	95% CI	*p* value
*Relative power*						
Delta	24.8	-2.1 to 51.6	.072	**33.3**	**6.7 to 59.9**	**.015**
Theta	**40.1**	**5.9 to 74.2**	**.022**	**48.6**	**13.6 to 83.7**	**.007**
Alpha1	-48.9	-100.6 to 2.6	.062	-38.3	-90.4 to 13.7	.147
Alpha2	-77.9	-175.9 to 20.0	.117	**-79.9**	-**154.1 to -5.6**	**.036**
Beta	**-27.9**	**-53.8 to -2.1**	**.035**	**-37.3**	**-62.8 to -11.7**	**.005**
Peak frequency	**-2.8**	**-4.7 to -0.70**	**.008**	**-2.7**	**-4.6 to -0.80**	**.006**

*CAMCOG* Cambridge Cognitive examination, *UPDRS-III* motor part of Unified Parkinson’s Disease Rating Scale, *95% CI* 95% confidence interval

**Fig. 4:**
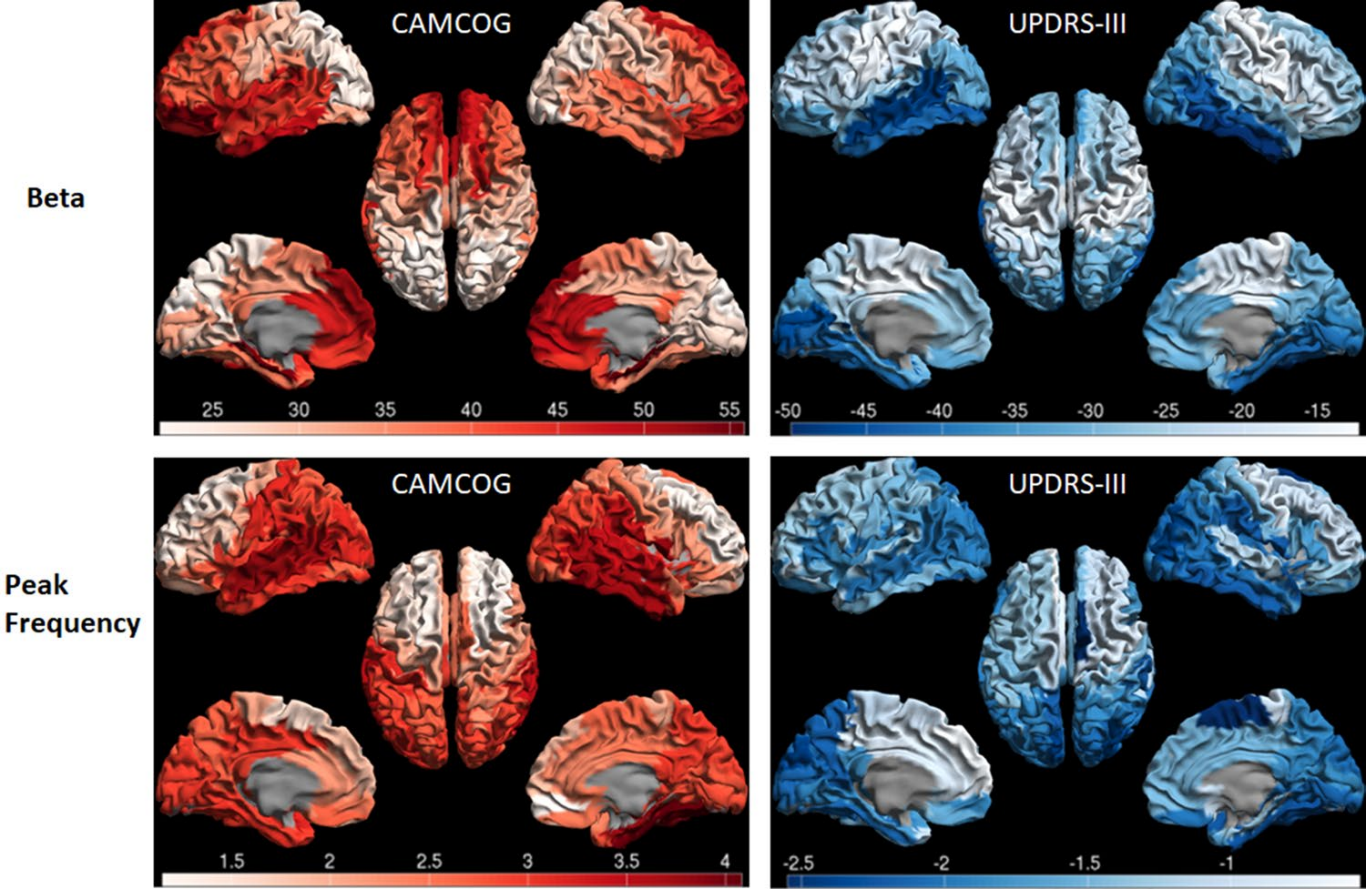
Topographic distribution of clinical associations. Distribution of longitudinal associations between relative beta band power and CAMCOG, relative beta band power and UPDRS-III, peak frequency and CAMCOG, peak frequency and UPDRS-III. Associations only concern cortical brain regions and are expressed as the estimated regression coefficient and displayed as a color-coded map on a parcellated template brain viewed from, in clockwise order, the left, top, right, right-midline and left-midline. Red represents a positive association, blue a negative association. Importantly, higher CAMCOG scores correspond to better cognitive functioning, whereas higher UPDRS-III scores correspond to worse motor functioning. Therefore, lower relative beta band power and lower peak frequency are associated with worse clinical (both cognitive and motor) functioning. All individual associations were statistically significant, except for a number of associations concerning the beta band (threshold around an estimated regression coefficient of -20). As we treated this analysis as a post-hoc analysis, we did not correct for multiple comparisons. The distribution of the remaining frequency bands that had significant associations with clinical measures of disease progression can be found in Supplementary Fig. 3

Next, for the spectral measures that demonstrated a significant association with a clinical measure, we displayed the cortical topographic distribution of (only) the significant associations as a post-hoc analysis. We restricted these figures to the cortical brain regions for visualization purposes, but we did calculate the individual associations of the subcortical brain regions (Supplementary Table 3). Figure 4 illustrates the longitudinal relations of relative beta band power and peak frequency with clinical measures of disease severity. The relationship between lower beta band power and worse cognitive performance was strongest for frontal cortical brain regions, whereas the relationship between lower beta band power and worse motor function was strongest (most negative) for the temporal-occipital brain regions. Interestingly, the relationship was weakest for the sensorimotor regions. For peak frequency, the strongest associations with clinical measures of disease severity were found for the temporal, parietal and occipital brain regions, both for the relationship with cognitive and motor performance. The remaining topographic distributions (delta-alpha2) can be found in Supplementary Fig. 3. Higher delta power in parieto-temporal regions was most strongly associated with cognitive and motor impairment (in the latter case also the occipital brain regions), higher frontal theta power with motor dysfunction, lower frontal alpha1 and alpha2 power with cognitive dysfunction, and lower parieto-temporal-occipital alpha2 power with motor impairment.

In Supplementary Table 3 we show the associations of individual subcortical brain regions with clinical measures of disease severity. The most outspoken pattern was the fact that activity in the hippocampus had the weakest association with CAMCOG and UPDRS-III for the frequency bands between delta and alpha2, but the strongest association with CAMCOG and UPDRS-III in the beta band. The results on peak frequency did not show a clear pattern.

## Discussion

In this longitudinal MEG study, we used a multiple longitudinal design to be able to analyze the changes in brain activity in Parkinson’s disease patients from disease onset up to a disease duration of 15–20 years. In the earliest Parkinson’s disease stages, we found activity changes in both cortical and subcortical brain regions, but, surprisingly, the changes in subcortical regions were less prominent than the changes in the cortical regions. Furthermore, in our longitudinal analyses we observed a progressive spectral slowing of brain activity throughout the course of the disease. This spectral slowing was strongly associated with clinical measures of disease progression (cognition and motor function), most outspoken for the cortical brain regions. We did not observe whole-brain functional connectivity changes in the baseline analysis and whole-brain functional connectivity hardly changed over time. However, in the connection-specific analysis we did observe significant changes at baseline (increased theta, decreased alpha2 functional connectivity), as well as over time in PD patients (increase in frontal delta and theta band functional connectivity).

The general pattern of spectral slowing involved both cortical and subcortical brain regions. Although we expected early functional changes in subcortical brain regions to be more outspoken than cortical changes, the subcortical changes were weaker than the cortical changes in case of alpha1 and beta power. Nonetheless, spectral power values were highly correlated between cortical and subcortical brain regions (Pearson’s *r* ranging between 0.913 and 0.965 for different frequency bands, *p* < 0.001). Besides a physiological explanation for this high correlation, as cortical and subcortical activity are structurally connected, field spread may have played a role, which is not corrected for in case of spectral analyses. Clearly, we do not know whether small subcortical changes may have preceded cortical changes at the premotor disease stage. Also, perhaps some of the subcortical changes were too local to be picked up by our MEG system, as the spatial resolution deeper in the brain is not as good as at the cortical level (Hillebrand and Barnes 2002). In addition, local (subcortical) pathological changes may have more distant (cortical) effects via ascending neurotransmitter systems. Spectral slowing at the cortical level is hypothesized to be a consequence of dopaminergic, noradrenergic, serotonergic and cholinergic dysfunction (Bosboom et al. 2009a; Détári et al. 1999; Rea et al. 2021) and could also be an effect of local Lewy body and tau pathology in thalamocortical circuits (Freunberger et al. 2011; Steriade et al. 1990). There are no previous EEG/MEG studies in early-stage Parkinson’s disease to compare our results with, but fMRI studies have shown functional changes (loss of functional connectivity) within the basal ganglia circuit in early-stage Parkinson’s disease (Rolinski et al. 2015; Szewczyk-Krolikowski et al. 2014) that were paralleled by whole-brain changes in activity (Pan et al. 2017).

We found strong longitudinal associations between spectral measures (spectral slowing) and clinical measures of disease severity. Based on the regression coefficients, we conclude that subcortical spectral measures were associated equally strongly with cognitive performance (CAMCOG) as cortical spectral measures (Table 2). However, unexpectedly, subcortical spectral measures were less strongly associated with motor function (UPDRS-III) than with cognitive performance (CAMCOG). Also, of the individual subcortical brain regions, relative beta band power of the hippocampus was most strongly associated with CAMCOG and UPDRS-III. Moreover, relative beta band power in the sensorimotor cortex was only poorly associated with UPDRS-III scores, compared to the rest of the cortex. The finding that both subcortical (except the hippocampus) spectral measures and beta band power in the sensorimotor cortex were associated poorly with UPDRS-III scores may originate from compensatory mechanisms secondary to motor impairment, as was previously hypothesized (Hirschmann et al. 2013; Pollok et al. 2013). Alternatively, the lack of strong associations may result from treatment with dopaminergic medication. The amount of dopaminergic medication (LEDD) is associated with lower relative beta power in the motor cortex (Pearson’s *r* left motor cortex, -0.213 *p* = 0.015, right motor cortex, -0.240 *p* = 0.005), but this could be both a disease effect or a treatment effect. Supplementary Fig. 4 demonstrates that sensorimotor beta power remains largely intact against a background of generalized slowing of cortical brain activity, especially between BL and FU1, despite worsening motor scores over time. For further considerations regarding the complex interplay between motor function and beta band power/functional connectivity we refer the reader to our review on this topic (Boon et al. 2019).

When considering the region-specific post-hoc analyses of the other frequency bands, we found that cognitive decline and motor dysfunction were related to spectral slowing, sometimes in the same brain regions (see for example peak frequency in Fig. 4), sometimes in different brain regions (see alpha2 in Supplementary Fig. 3). As hypothesized, lower peak frequency in posterior cortical brain regions correlated strongest with global cognitive decline (Fig. 4). This may reflect ‘posterior cortical dysfunction’, a clinical profile that may indicate a higher risk of developing PD dementia (Kehagia et al. 2013).

A correlation of oscillatory slowing with motor dysfunction has only been reported incidentally (Morita et al. 2009) and has not been found in other studies (Geraedts et al. 2018; Olde Dubbelink et al. 2013a). Possibly, our long follow-up duration allowed us to find the correlations between spectral slowing and motor dysfunction. Motor and cognitive impairment may be associated with spectral slowing via a general underlying mechanism. When we added UPDRS-III performance as a covariate in the linear mixed-model for the association between CAMCOG and peak frequency, the association remained present (standardized effect size 2.32, *p* = 0.001), but not the other way around (standardized effect size -1.50, *p* = 0.128). We therefore conclude that, although motor and cognitive impairment are both related to the same underlying disease process, their pathophysiology does not fully overlap.

We chose not to include functional connectivity in our analyses of longitudinal associations with clinical measures of disease severity. At the whole-brain baseline analysis, we did not observe significant group differences in functional connectivity and over time, only subcortical beta band functional connectivity significantly changed. This was unexpected, as a previous analysis in the same cohort showed longitudinal decreases in alpha1 and alpha2 functional connectivity, although only between baseline and FU1 and with another measure of functional connectivity (Olde Dubbelink et al. 2013b). In addition, a recent high-density EEG study with a longitudinal design demonstrated a progressive loss of functional connectivity in correlation with global cognitive decline and lateralization of motor symptoms (Yassine et al. 2022). The differences between the present and previous study may partly be explained by the fact that AEC-c is an amplitude-based measure that is fundamentally different from the phase-based measures (phase lag index, phase locking value) that were used in other longitudinal studies on PD patients (Olde Dubbelink et al. 2013b; Yassine et al. 2022).

When we additionally performed a connection-specific analysis, we did observe local differences in functional connectivity. We found higher theta band functional connectivity of frontal connections and lower alpha2 band functional connectivity of parieto-occipital connections in PD patients compared with HCs. The loss of alpha2-band functional connectivity in PD patients is in line with previous studies in PD (Olde Dubbelink et al. 2013b; Yassine et al. 2022), although in these studies the changes were present throughout the brain. The parieto-occipital changes in alpha2 functional connectivity match the observations in Alzheimer’s disease (AD), in which the same (amplitude-based) functional connectivity measure was used (Schoonhoven et al. 2022). In addition, in this latter study, higher delta (AEC-d) and theta band (phase lag index) functional connectivity was seen in AD patients, which may reflect a general tendency to find higher functional connectivity values for slower frequencies in neurodegenerative diseases. In our connection-specific analysis, we found higher frontal theta band functional connectivity in PD patients, both at baseline and during follow-up.

The results of the connection-specific analysis indicate that potentially important findings can be missed in a whole-brain analysis in which subtle differences may be ‘averaged out’. Furthermore, the addition of a third time point and a different MEG system may have confounded the analysis. The only significant whole-brain change we observed was an increase in beta band functional connectivity between FU1 and FU2. Since an increase in beta band functional connectivity was also present in our healthy control group, this may be related to the change from one MEG system to the other (Boon et al. 2021).

Our results confirm that neurophysiological patterns are good candidates in the search for biomarkers of the degenerative process in Parkinson’s disease. Although we did not study the risk of conversion to Parkinson’s disease dementia, a previous analysis in this study cohort demonstrated that lower beta band power, especially in the posterior brain regions, was a strong predictor for conversion (Olde Dubbelink et al. 2014b). Our result in Fig. 4 was therefore somewhat surprising, as we found that beta band power of frontal cortical brain regions correlated strongest with global cognitive decline. The identification of a subgroup of Parkinson’s disease patients at high risk for dementia may be important for patients and caregivers in the context of advanced care planning, and also for future studies aimed at disease-modifying therapies to slow down cognitive decline. In the latter case, a neurophysiological biomarker may also serve as an objective read-out parameter of treatment success.

A strength of the current analysis is the multiple longitudinal design with a long follow-up duration of seven years, which allowed us to longitudinally cover 15–20 years of the disease course. In addition, the inclusion of a third time point (second follow-up) adds robustness to previously published results on spectral power at the first two time points (Olde Dubbelink et al. 2013a), as it confirms that the observed trends in spectral slowing are progressive over time. In addition, this is the first MEG study exploring the presence of subcortical neurophysiological changes in the earliest clinical motor stages of Parkinson’s disease, as well as the further development of these changes over time. Three potential limitations of our study deserve consideration. First, our baseline cohort decreased from 61 patients at BL to 39 patients at FU1. This is explained by the fact that data of 19 patients could not be used (low quality data, no MRI-scan; see also Fig. 1). 23 patients were lost to follow-up 2, possibly due to high disease burden. Given that this group was clinically more severely affected, the dropout of these subjects can only have led to an underestimation of true disease effects. In addition, our linear mixed models account for missing data, so that reliable longitudinal associations could still be established. Second, several subjects could not be included in the follow-up visits because they had poor-quality data or because they were lost to follow-up (see Fig. 1 for an overview). The majority of missing data was due to poor-quality MEG or MRI data, which is a random phenomenon. We do not have an overview of the reasons why patients were lost to follow-up, but there may have been a selective dropout of patients with a high (subjective) disease burden. However, this could only have led to an underestimation of the true effects.

Third, although there is increasing evidence that projecting MEG to subcortical sources is feasible, MEG is most sensitive to cortical sources (Hillebrand and Barnes 2002). The sensitivity for subcortical sources can be further improved using new analysis techniques such as ‘blind source separation’ (López-Madrona et al. 2022; Pizzo et al. 2019), methods that increase the contrast between cortical and subcortical sources (Quraan et al. 2011), or in-mouth sensors (Tierney et al. 2021).

In conclusion, already at the earliest disease stages of Parkinson’s disease, there are neurophysiological changes in Parkinson’s disease patients both at the subcortical and cortical level, most outspoken for the latter. In our analysis using a multiple longitudinal design, spanning 15–20 years of disease duration, we found strong longitudinal associations between spectral slowing of brain activity and clinical measures of disease severity, especially cognition. Functional connectivity changes were present, but only localized and not at the whole-brain level. Our results indicate that spectral power is a promising candidate in the search for a non-invasive marker to monitor the ongoing disease process in Parkinson’s disease.

## Supplementary Information

Below is the link to the electronic supplementary material.

Supplementary file1 (DOCX 3045 kb)
