# Supplementary material for: Cortical and Subcortical Changes in MEG Activity Reflect Parkinson’s Progression over a Period of 7 Years
Source: Brain Topogr. 2023 May 8;36(4):566–80. doi: 10.1007/s10548-023-00965-w (PMC10293384; doi:10.1007/s10548-023-00965-w)
Supplement: Supplementary file 1 — Supplementary file1 (DOCX 3045 kb) [file 10548_2023_965_MOESM1_ESM.docx]

**Supplementary Materials**

**Supplementary Table 1**

AAL atlas, with regions ordered as in Gong et al..(Gong, He et al. 2009) Regions in bold were grouped as subcortical brain regions (region 79-90).

|  | **Left hemisphere** |  | **Right hemisphere** |
| --- | --- | --- | --- |
| 1 | Gyrus rectus | 40 | Gyrus rectus |
| 2 | Olfactory cortex | 41 | Olfactory cortex |
| 3 | Super frontal gyrus, orbital part | 42 | Super frontal gyrus, orbital part |
| 4 | Superior frontal gyrus, medial orbital part | 43 | Superior frontal gyrus, medial orbital part |
| 5 | Middle frontal gyrus, orbital part | 44 | Middle frontal gyrus, orbital part |
| 6 | Inferior frontal gyrus, orbital part | 45 | Inferior frontal gyrus, orbital part |
| 7 | Superior frontal gyrus, dorsolateral part | 46 | Superior frontal gyrus, dorsolateral part |
| 8 | Middle frontal gyrus | 47 | Middle frontal gyrus |
| 9 | Inferior frontal gyrus, opercular part | 48 | Inferior frontal gyrus, opercular part |
| 10 | Inferior frontal gyrus, triangular part | 49 | Inferior frontal gyrus, triangular part |
| 11 | Superior frontal gyrus, medial part | 50 | Superior frontal gyrus, medial part |
| 12 | Supplementary motor area | 51 | Supplementary motor area |
| 13 | Paracentral lobule | 52 | Paracentral lobule |
| 14 | Precentral gyrus | 53 | Precentral gyrus |
| 15 | Rolandic operculum | 54 | Rolandic operculum |
| 16 | Postcentral gyrus | 55 | Postcentral gyrus |
| 17 | Superior parietal gyrus | 56 | Superior parietal gyrus |
| 18 | Inferior parietal gyrus | 57 | Inferior parietal gyrus |
| 19 | Supramarginal gyrus | 58 | Supramarginal gyrus |
| 20 | Angular gyrus | 59 | Angular gyrus |
| 21 | Precuneus | 60 | Precuneus |
| 22 | Superior occipital gyrus | 61 | Superior occipital gyrus |
| 23 | Middle occipital gyrus | 62 | Middle occipital gyrus |
| 24 | Inferior occipital gyrus | 63 | Inferior occipital gyrus |
| 25 | Calcarine fissure and surrounding cortex | 64 | Calcarine fissure and surrounding cortex |
| 26 | Cuneus | 65 | Cuneus |
| 27 | Lingual gyrus | 66 | Lingual gyrus |
| 28 | Fusiform gyrus | 67 | Fusiform gyrus |
| 29 | Heschl gyrus | 68 | Heschl gyrus |
| 30 | Superior temporal gyrus | 69 | Superior temporal gyrus |
| 31 | Middle temporal gyrus | 70 | Middle temporal gyrus |
| 32 | Inferior temporal gyrus | 71 | Inferior temporal gyrus |
| 33 | Temporal pole: superior temporal gyrus | 72 | Temporal pole: superior temporal gyrus |
| 34 | Temporal pole: middle temporal gyrus | 73 | Temporal pole: middle temporal gyrus |
| 35 | Parahippocampal gyrus | 74 | Parahippocampal gyrus |
| 36 | Anterior (para)cingulate gyrus | 75 | Anterior (para)cingulate gyrus |
| 37 | Median (para)cingulate gyrus | 76 | Median (para)cingulate gyrus |
| 38 | Posterior cingulate gyrus | 77 | Posterior cingulate gyrus |
| 39 | Insula | 78 | Insula |
| **79** | **Hippocampus** | **80** | **Hippocampus** |
| **81** | **Amygdala** | **82** | **Amygdala** |
| **83** | **Caudate nucleus** | **84** | **Caudate nucleus** |
| **85** | **Putamen** | **86** | **Putamen** |
| **87** | **Pallidum** | **88** | **Pallidum** |
| **89** | **Thalamus** | **90** | **Thalamus** |

**Color - Supplementary Figure 1** Overview of the statistical analysis using linear mixed models

The blue dotted line indicates a longitudinal analysis using linear mixed models. The blue dotted box indicates a longitudinal association between a neurophysiological measure (blue) and a clinical measure of disease severity (red, motor function; green, cognitive function).

**
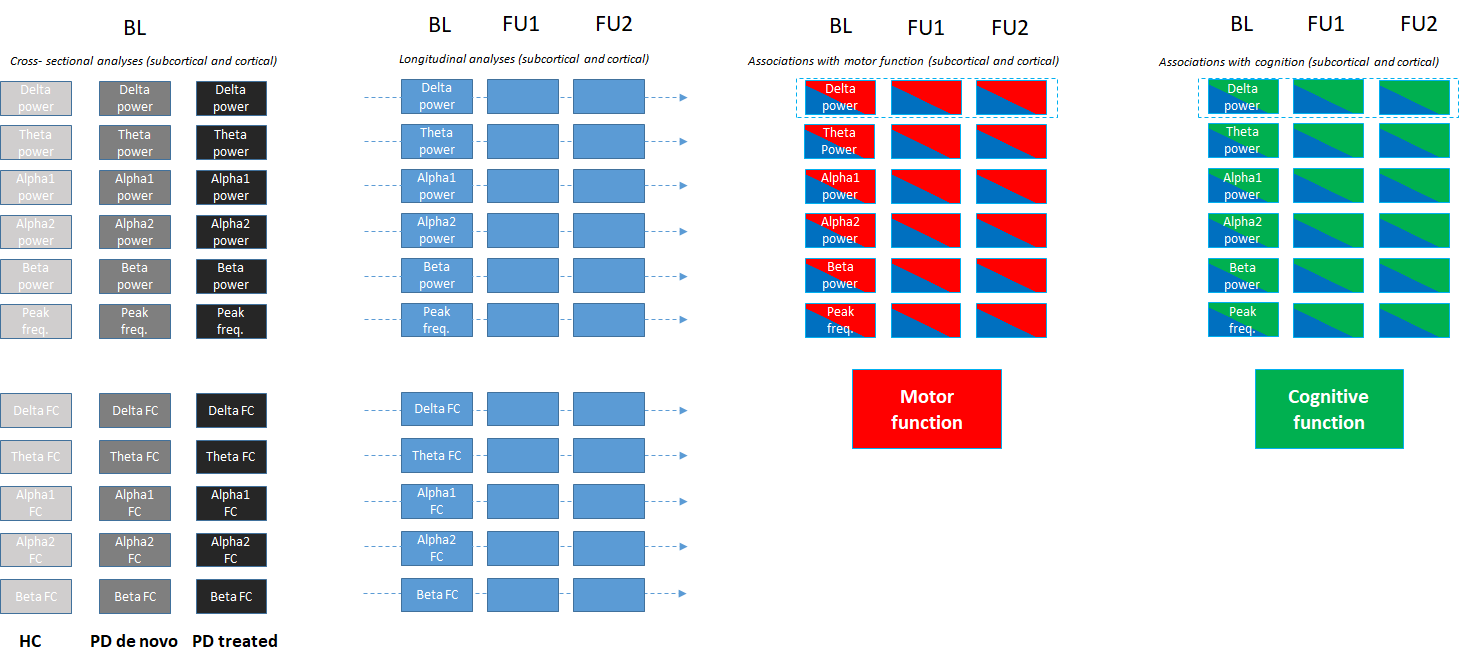
**

**Supplementary Table 2** Longitudinal changes in spectral measures

|  | **BL-FU1** | | | **FU1-FU2** | | | **BL-FU2** | | |
| --- | --- | --- | --- | --- | --- | --- | --- | --- | --- |
|  | Estimated regression coefficient | 95% CI | *p* value | Estimated regression coefficient | 95% CI | *p* value | Estimated regression coefficient | 95% CI | *p* value |
| *Subcortical brain regions* |  |  |  |  |  |  |  |  |  |
| Delta power | 0.018 | -0.009 to 0.046 | .187 | 0.029 | -0.002 to 0.059 | .060 | **0.047** | **0.010 to 0.083** | **.008** |
| Theta power | 0.019 | 0.004 to 0.035 | .017 | 0.017 | -0.001 to 0.337 | .053 | **0.035** | **0.016 to 0.058** | **.001** |
| Alpha1 power | -0.011 | -0.020 to -0.001 | .041 | 0.002 | -0.010 to 0.014 | .687 | -0.009 | -0.025 to 0.005 | .217 |
| Alpha2 power | -0.004 | -0.011 to 0.002 | .267 | -0.007 | -0.015 to -0.001 | .049 | -0.011 | -0.020 to -0.003 | .013 |
| Beta power | -0.021 | -0.044 to 0.001 | .059 | **-0.040** | **-0.060 to -0.017** | **.001** | **-0.061** | **-0.093 to -0.032** | **< .001** |
| Peak frequency | **-0.459** | **-0.785 to -0.182** | **.002** | -0.182 | -0.398 to -0.038 | .259 | **-0.640** | **-1.09 to -0.277** | **.001** |
|  |  |  |  |  |  |  |  |  |  |
| *Cortical brain regions* |  |  |  |  |  |  |  |  |  |
| Delta power | 0.020 | -0.005 to 0.045 | .115 | **0.065** | **0.038 to 0.092** | **< .001** | **0.085** | **0.052 to 0.119** | **< .001** |
| Theta power | **0.022** | **0.007 to 0.037** | **.003** | 0.000 | -0.015 to 0.015 | .995 | 0.022 | 0.002 to 0.042 | 0.030 |
| Alpha1 power | -0.009 | -0.020 to 0.002 | .117 | -0.004 | -0.016 to 0.008 | .525 | -0.013 | -0.018 to 0.002 | 0.097 |
| Alpha2 power | -0.008 | -0.015 to -0.001 | .048 | **-0.011** | **-0.019 to -0.003** | **.006** | **-0.019** | **-0.029 to -0.009** | **< .001** |
| Beta power | -0.025 | -0.044 to -0.006 | .012 | **-0.051** | **-0.071 to -0.031** | **< .001** | **-0.076** | **-0.049 to -0.103** | **< .001** |
| Peak frequency | **-0.429** | **-0.717 to -0.168** | **.004** | -0.247 | -0.555 to -0.062 | .116 | **-0.676** | **-1.07 to -0.283** | **.001** |

Overview of the longitudinal changes, separately for each frequency band and for each group of brain regions (subcortical and cortical). Statistics were performed using linear mixed-models. We applied a p-value threshold of 0.05/6 for significance (Bonferroni correction). Significant results are indicated in bold.

**Supplementary Figure 2** Functional connectivity

**A) Baseline: Healthy controls, de novo, and more advanced treated PD patients**


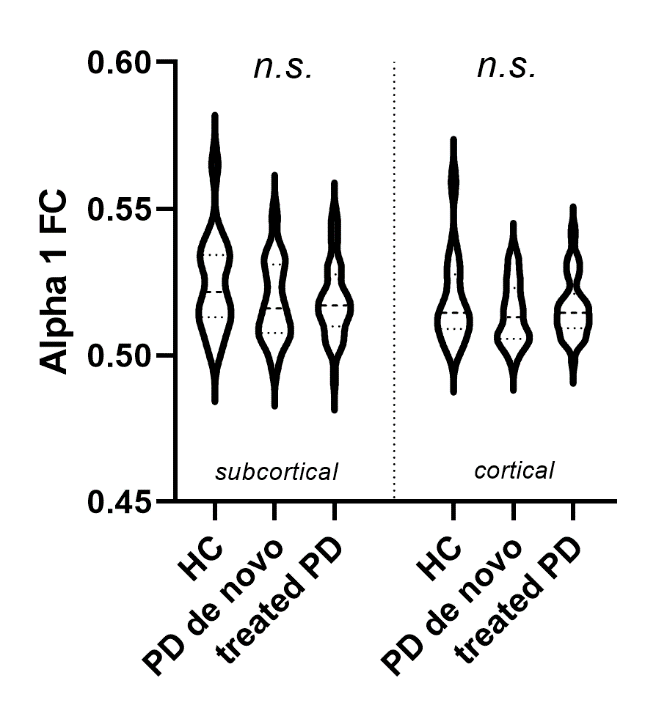

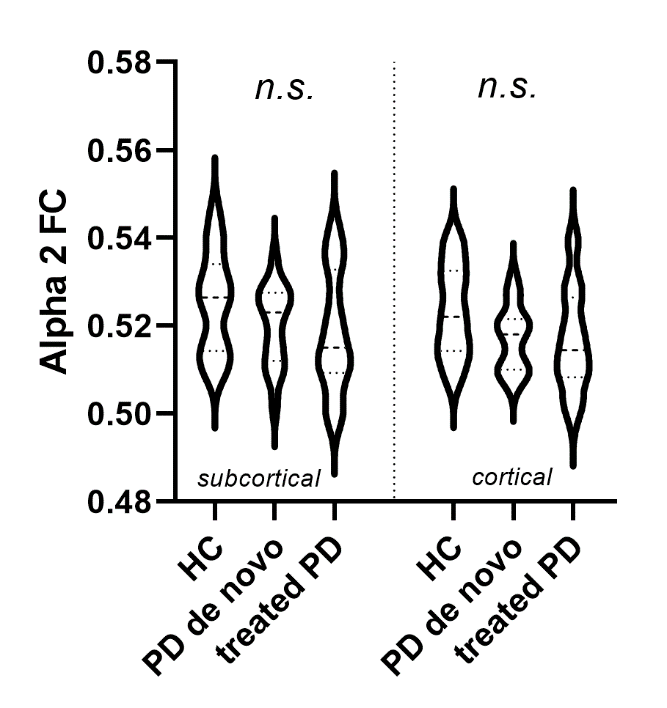

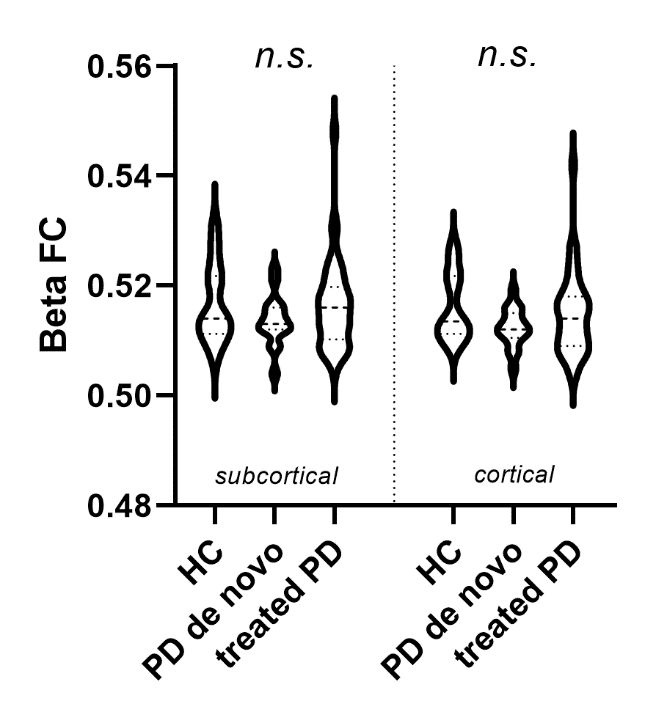

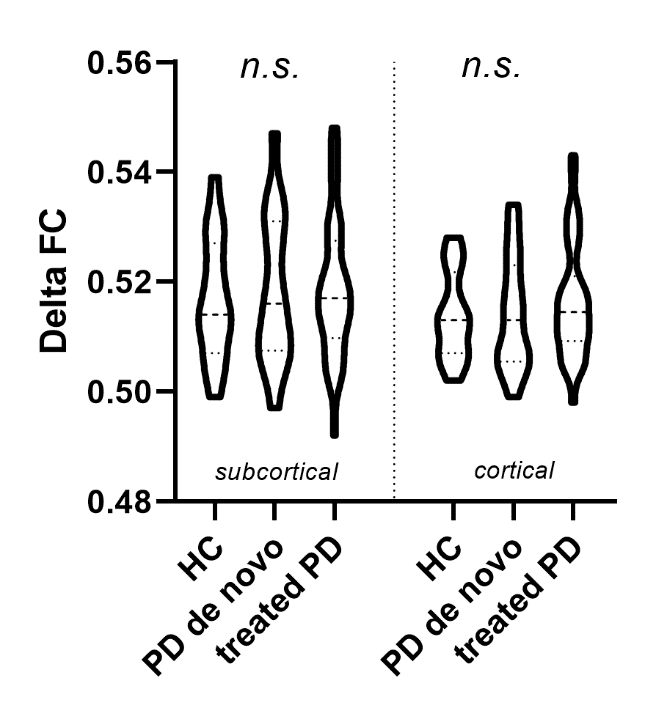

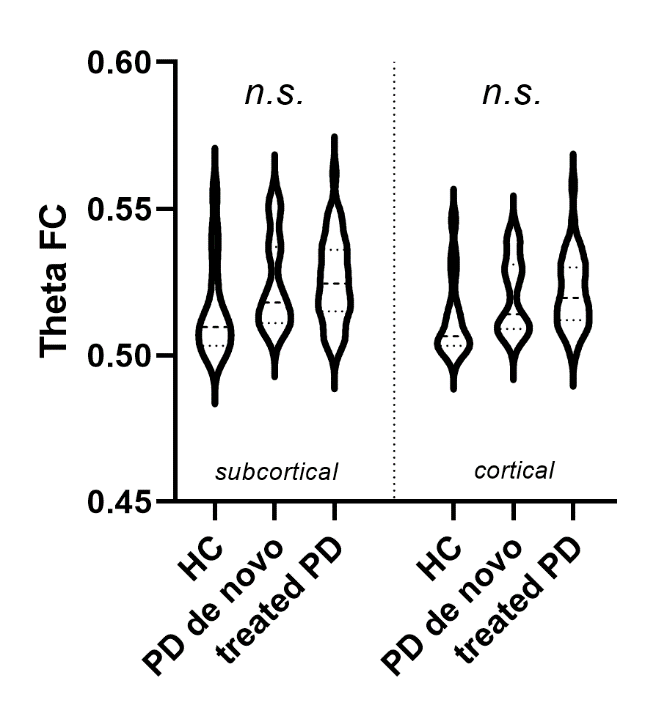


**B) Longitudinal changes**


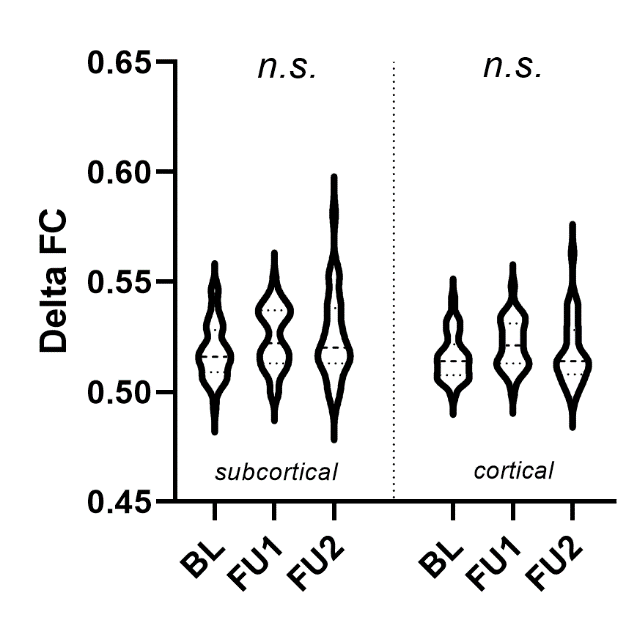

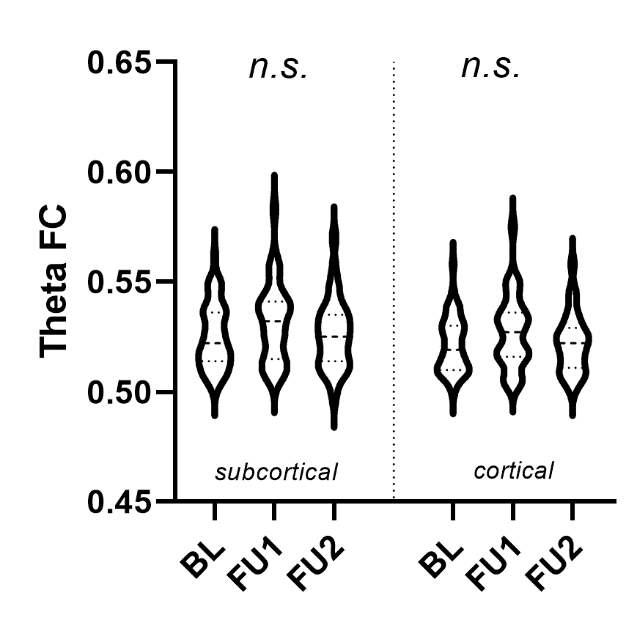

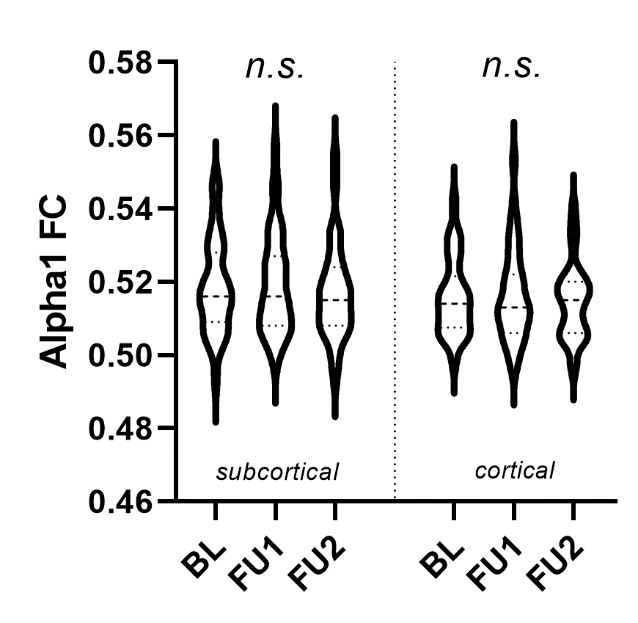

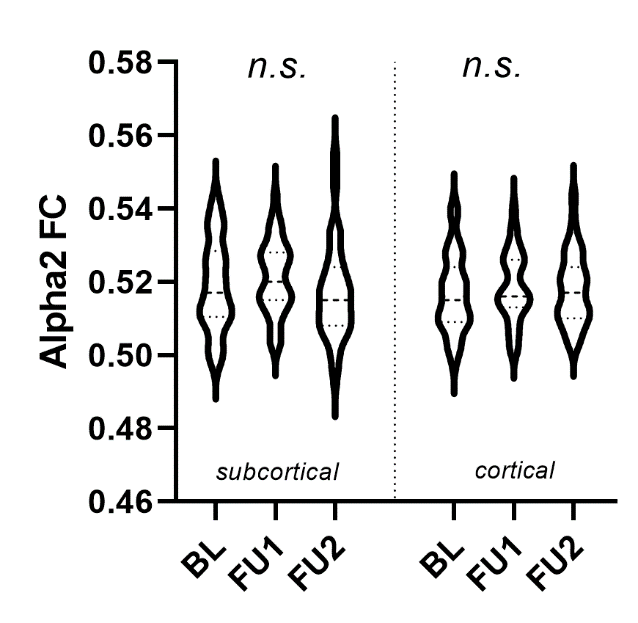

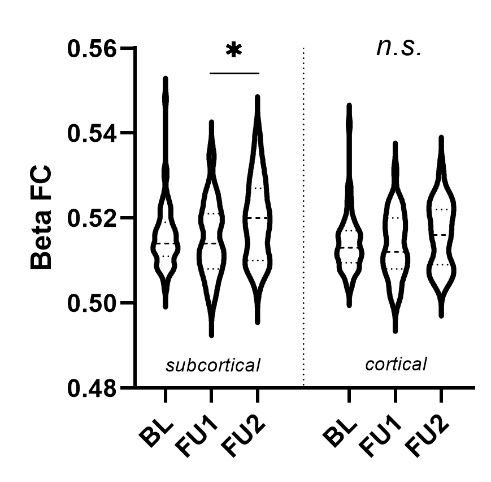


1. Violin plots summarizing the baseline comparison between three groups; Healthy controls (HC), ‘de novo’ untreated PD patients, and more advanced treated PD patients. Linear-mixed models were performed and a significance level of 0.05/6 (Bonferroni correction) was applied.
2. Violin plots summarizing the longitudinal analysis. Statistical testing was performed using linear mixed-models with a significance level of 0.05/6.

n.s., non-significant; FU1, follow-up 1; FU2, follow-up 2


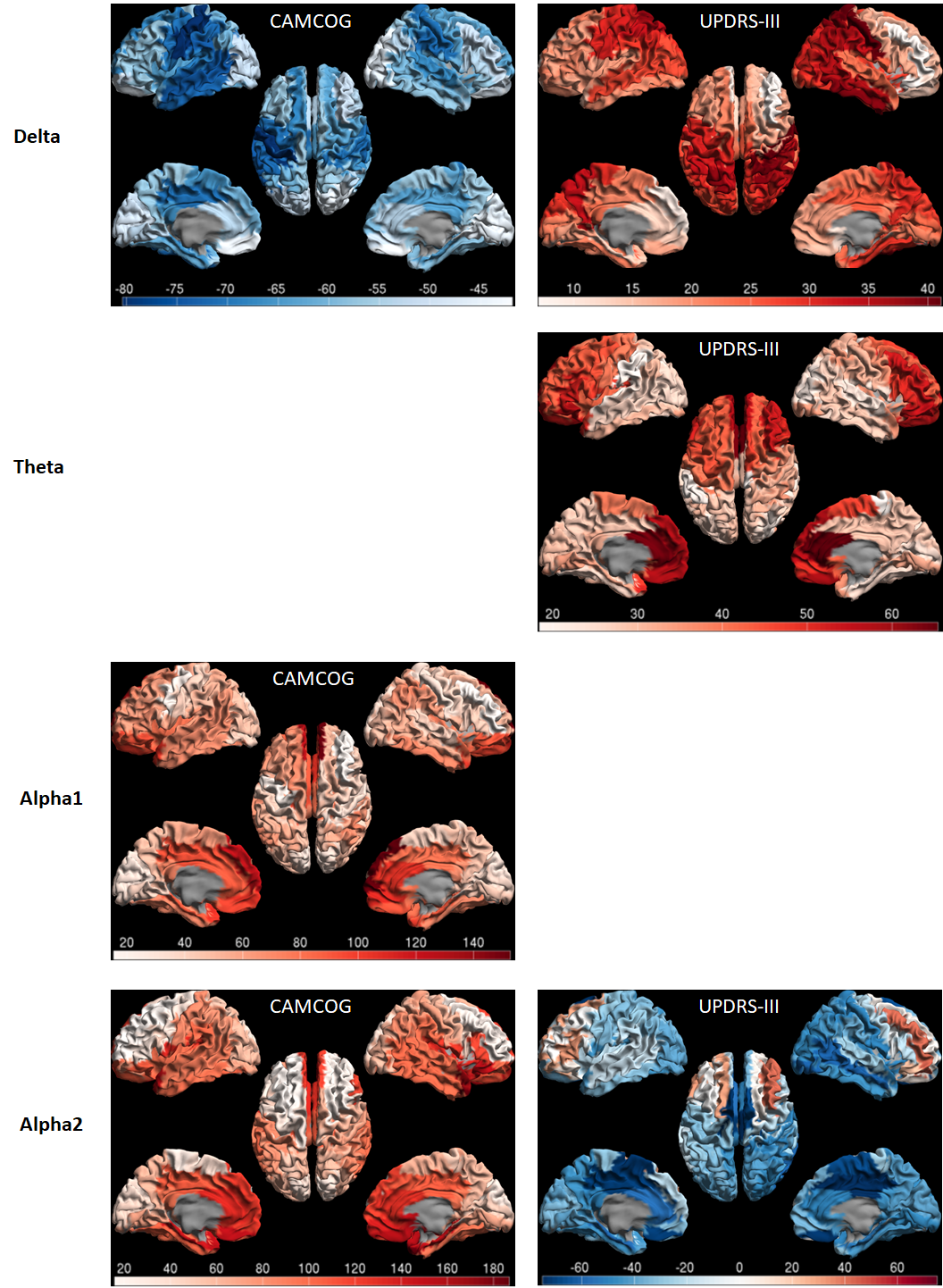


**Color - Supplementary Figure 3**

Post-hoc analysis analyzing the distribution of longitudinal associations between relative delta-alpha2 band power of the cortical brain regions and CAMCOG (left column) and UPDRS-III (right column). Post-hoc visualization was only performed for the frequency bands that had a significant association with the clinical measure (CAMCOG or UDPRS-III).

Associations are expressed as the estimated regression coefficient and displayed as a color-coded map on a parcellated template brain viewed from, in clockwise order, the left, top, right, right-midline and left-midline.

**Supplementary Table 3**

Post-hoc analyses analyzing the distribution of longitudinal associations between relative spectral power (delta-beta band) and peak frequency of the subcortical brain regions with A) CAMCOG B) UPDRS-III. The post-hoc analyses were only performed for the frequency bands that had a significant association with the clinical measure.

R/L; right/left

| **CAMCOG** | **Estimated regression coefficients** | | | |  |
| --- | --- | --- | --- | --- | --- |
| **AAL region** | **Delta** | **Alpha1** | **Alpha2** | **Beta** | **Peak frequency** |
| 79 Hippocampus L | -62.5 | 66.3 | 128.7 | 52.8 | 3.1 |
| 80 Hippocampus R | -60.9 | 73.2 | 128.3 | 54.7 | 3.3 |
| 81 Amygdala L | -67.2 | 100.0 | 146.2 | 49.9 | 3.0 |
| 82 Amygdala R | -67.0 | 90.8 | 190.0 | 46.6 | 3.3 |
| 83 Caudate nucleus L | -65.7 | 92.3 | 109.0 | 44.0 | 2.5 |
| 84 Caudate nucleus R | -64.6 | 86.9 | 151.7 | 45.9 | 2.8 |
| 85 Putamen L | -68.8 | 80.3 | 126.0 | 45.0 | 2.8 |
| 86 Putamen R | -62.4 | 87.2 | 156.8 | 43.3 | 3.7 |
| 87 Pallidum L | -69.4 | 81.5 | 143.4 | 45.0 | 2.8 |
| 88 Pallidum R | -66.9 | 90.1 | 186.0 | 47.2 | 3.4 |
| 89 Thalamus L | -63.9 | 73.4 | 125.6 | 36.2 | 2.7 |
| 90 Thalamus R | -66.7 | 80.4 | 150.2 | 39.7 | 3.2 |

| **UPDRS-III** | **Estimated regression coefficients** | | |
| --- | --- | --- | --- |
| **AAL region** | **Theta** | **Beta** | **Peak frequency** |
| 79 Hippocampus L | 27.6 | -32.7 | -2.1 |
| 80 Hippocampus R | 23.8 | -40.9 | -1.6 |
| 81 Amygdala L | 39.5 | -32.2 | -1.9 |
| 82 Amygdala R | 29.5 | -26.0 | -1.8 |
| 83 Caudate nucleus L | 47.0 | -26.0 | -1.8 |
| 84 Caudate nucleus R | 45.2 | -27.2 | -2.1 |
| 85 Putamen L | 40.9 | -22.9 | -2.4 |
| 86 Putamen R | 35.5 | -20.1 | -2.4 |
| 87 Pallidum L | 35.4 | -22.5 | -2.2 |
| 88 Pallidum R | 34.7 | -20.6 | -2.0 |
| 89 Thalamus L | 30.0 | -16.1 | -2.3 |
| 90 Thalamus R | 38.4 | -25.2 | -2.3 |

**Color - Supplementary Figure 4**

Relative beta band power at baseline (healthy controls and PD), follow-up 1 and follow-up 2. Power values are displayed as a color-coded map on a parcellated template brain viewed from, in clockwise order, the left, top, right, right-midline and left-midline. Note that sensorimotor beta power remains largely intact on top of a general cortical slowing, especially at baseline (i.e. compare PD and HC at baseline).


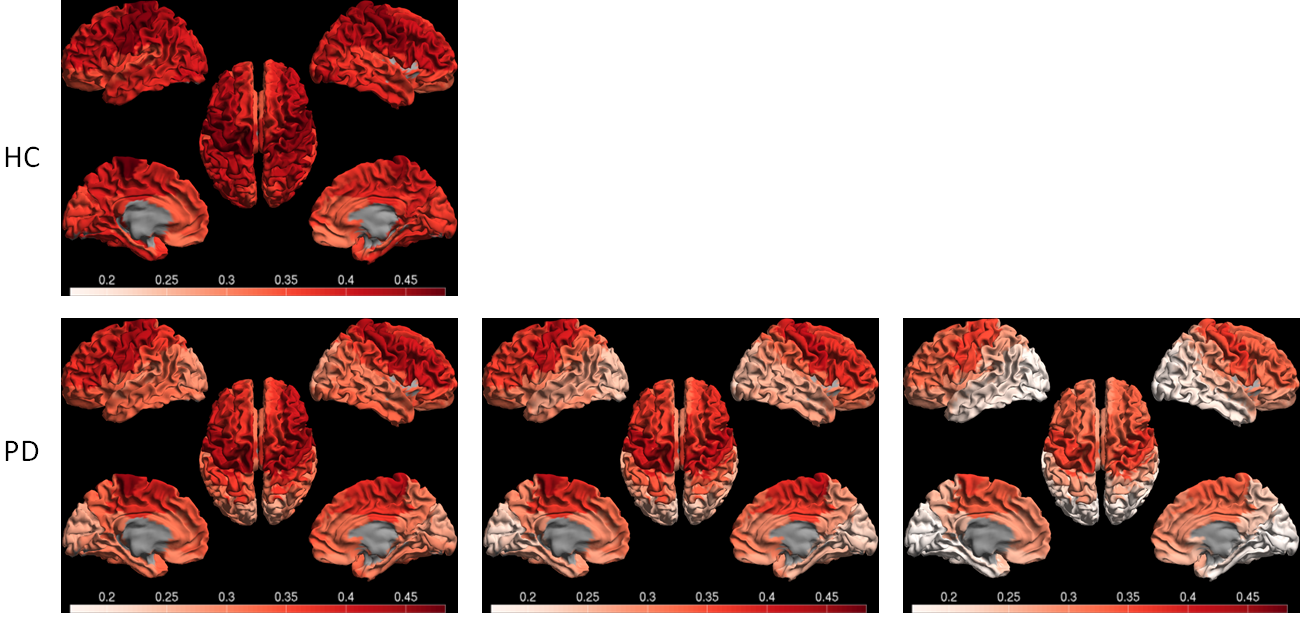


**References**

Gong, G., Y. He, L. Concha, C. Lebel, D. W. Gross, A. C. Evans and C. Beaulieu (2009). "Mapping anatomical connectivity patterns of human cerebral cortex using in vivo diffusion tensor imaging tractography." Cereb Cortex **19**(3): 524-536.
